# A Case of Euglycemic Diabetic Ketoacidosis Associated With a Sodium-Glucose Cotransporter-2 (SGLT2) Inhibitor

**DOI:** 10.7759/cureus.75399

**Published:** 2024-12-09

**Authors:** Antoine Hedary, Lacie Melder, Micah Pippin

**Affiliations:** 1 Family Medicine, Louisiana State University Health Sciences Center, Alexandria, USA

**Keywords:** diabetes mellitus type 2, diabetes treatment, diabetic ketoacidosis (dka), precipitating factors for euglycemic dka, sodium-glucose cotransporter-2 (sglt2) inhibitors

## Abstract

Euglycemic diabetic ketoacidosis is a rare metabolic derangement seen in both type 1 and type 2 diabetes. Initially characterized decades ago, the prevalence of euglycemic diabetic ketoacidosis has increased in recent years following the introduction of sodium-glucose cotransporter-2 (SGLT2) inhibitors. Here, we present a case of euglycemic diabetic ketoacidosis associated with SGLT2 inhibitors.

## Introduction

Diabetic ketoacidosis is a well-established metabolic derangement classically characterized by the triad of hyperglycemia, elevated anion gap metabolic acidosis, and ketonemia [1-2]. Initial symptoms may include nausea, vomiting, abdominal pain, and poor appetite, which may progress to confusion, metabolic encephalopathy, and coma as the metabolic derangements become more severe [1-4]. Diabetic ketoacidosis is more commonly seen in patients with type 1 diabetes rather than type 2 diabetes. It occurs when insulin deficiency leads to uncontrolled hyperglycemia with decreased intracellular glucose metabolism; this initiates lipolysis, and the continuous buildup of ketones leads to an acidotic state [1-2].

Diabetic ketoacidosis often occurs as a result of metabolic stress, such as acute illness, infection, starvation, alcohol use, or pregnancy [2-4]. These same factors may trigger diabetic ketoacidosis in patients on sodium-glucose cotransporter-2 (SGLT2) inhibitors, even in the absence of abnormally elevated blood glucose levels [2-3]. However, the pathophysiology in these cases is unique compared to classic diabetic ketoacidosis.

The absence of hyperglycemia in a patient with diabetic ketoacidosis presents a diagnostic challenge. The following case illustrates euglycemic diabetic ketoacidosis in a patient on an SGLT2 inhibitor.

## Case presentation

A 77-year-old female with a past medical history of hypertension, non-insulin-dependent type 2 diabetes, coronary artery disease on dual antiplatelet therapy, and carotid stenosis presented to the emergency department after a fall. On admission, the patient complained of feeling ill for several days leading up to the fall. The fall occurred at home, and the patient reported feeling dizzy and losing consciousness before falling. The patient arrived as a transfer from an outside emergency department, where the reading of a computed tomography (CT) scan of the head suggested that the patient had sustained a trace subdural hematoma without mass effect. Systolic blood pressure readings in the 80s mmHg were reported from the transferring facility, and the patient was given a bolus of 2 L of normal saline prior to transfer.

**Figure 1:**
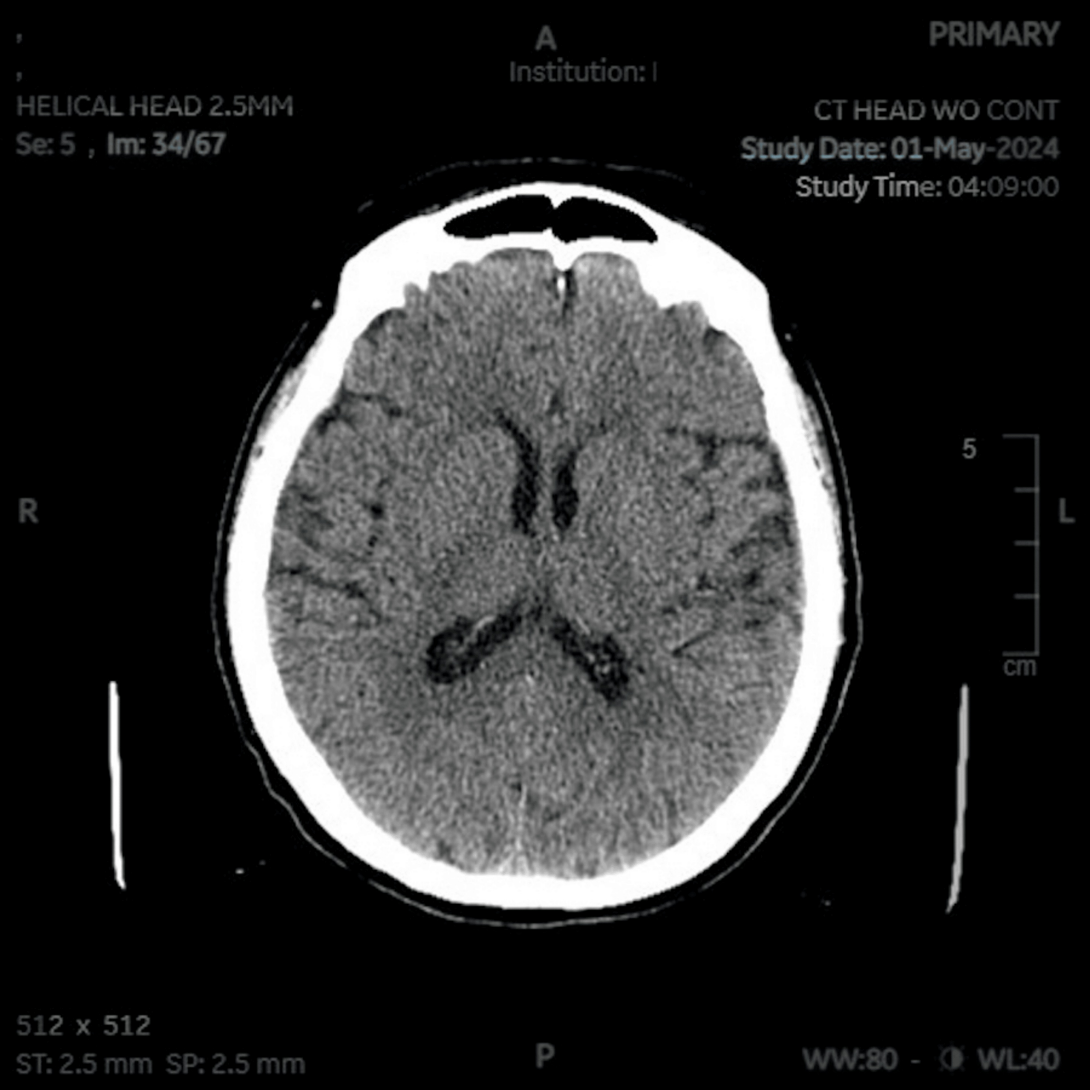
CT of the head with axial view demonstrating no subdural hematoma

**Figure 2:**
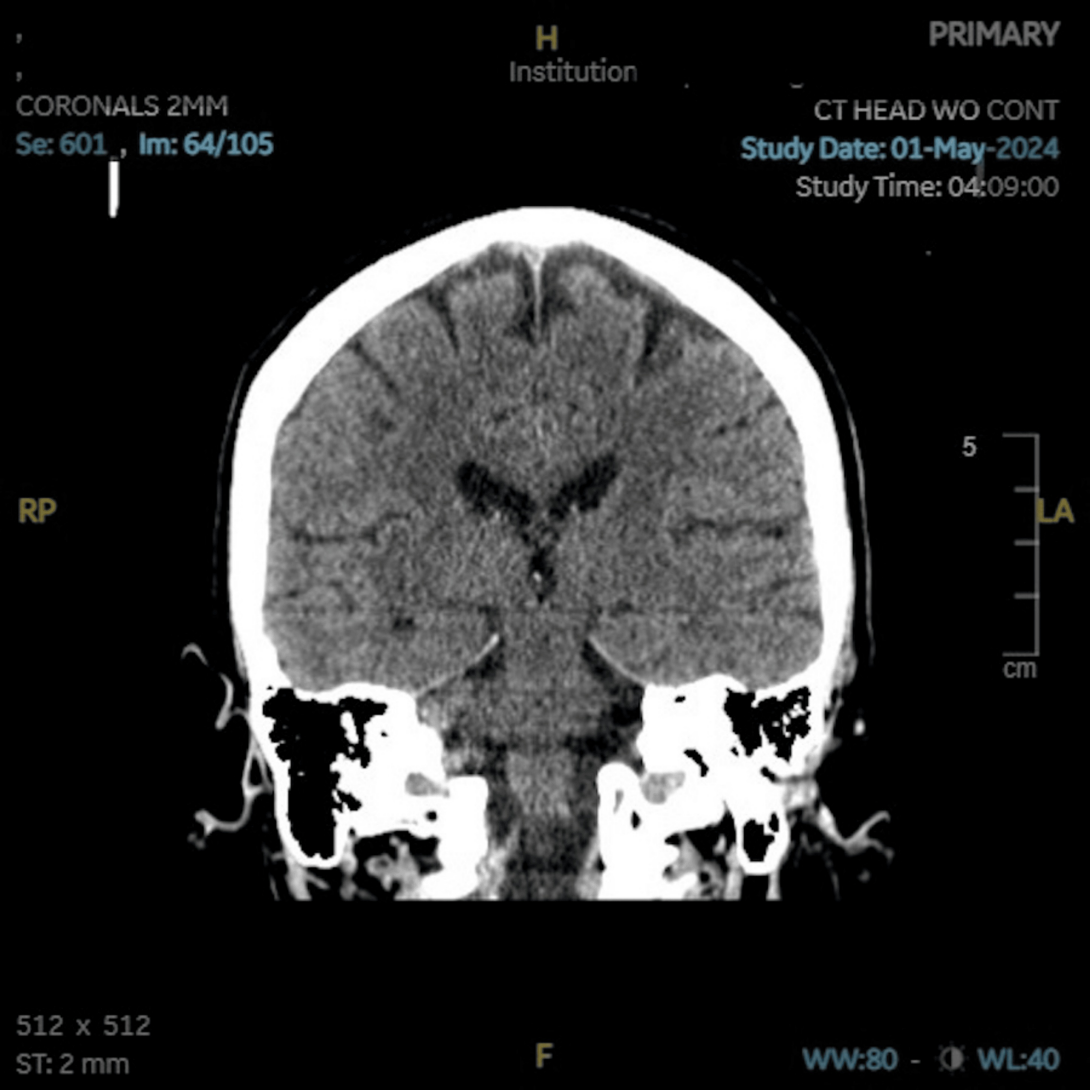
CT of the head with coronal view demonstrating no subdural hematoma

On presentation, the patient appeared without signs of confusion or encephalopathy and was alert and oriented to person, place, and time. The patient reported taking long-acting insulin, sitagliptin, metformin, and dapagliflozin at home for diabetes. Vital signs on admission were initially non-concerning: oxygen saturation on pulse oximetry was 100% on room air, blood pressure of 120/56 mmHg with a mean arterial pressure of 77 mmHg, temperature of 98.1°F, heart rate of 76 beats per minute, and respiratory rate of 18 breaths per minute. The physical exam was remarkable for a hypovolemic-appearing patient with trauma to the posterior scalp and ecchymoses on the upper extremities but otherwise unremarkable neurologically. The patient complained of moderate, intermittent nausea and vomiting, which were initially attributed to concussion symptoms. A repeat head CT scan did not demonstrate any subdural hematoma, only minimal atrophy and age-related changes (Figures 1-2).

The trauma and neurosurgery teams were consulted upon arrival at the emergency department, and both independently recommended against any immediate surgical intervention. The patient was admitted to the intensive care unit (ICU) for close observation per neurosurgery's recommendations. Initial laboratory studies revealed a slightly elevated blood glucose level of 135 mg/dL (normal 70-120 mg/dL), slightly elevated chloride of 109 mmol/L (normal 98-107 mmol/L), significantly low bicarbonate of less than seven mmol/L (normal 21-32 mmol/L), plasma creatinine of 1.1 mg/dL (normal 0.5-1.1 mg/dL), decreased estimated glomerular filtration rate (eGFR) of 48 mL/min/1.73 m^2^ (normal >90 mL/min/1.73 m^2^), and an anion gap of 28 (normal 0-15) (Table 1).

**Table 1:** Initial laboratory assessment

Laboratory testing of serum		
Labs	Patient values	Reference values
White blood cells (WBC) (cells/mm^3^)	8.7	5.0-10.0
Hemoglobin (gm/dL)	13.0	12.0-16.0
Hematocrit (%)	42.5	35.0-49.0
Sodium (mmol/L)	140	135-148
Potassium (mmol/L)	4.4	3.3-5.1
Chloride (mmol/L)	109	98-107
Serum carbon dioxide (CO_2_) (mmol/L)	<7	21-32
Urea nitrogen (mg/dL)	14	6-19
Creatinine (mg/dL)	1.10	0.50-1.10
Serum glucose (mg/dL)	135	70-120
Calcium (mg/dL)	7.7	8.4-10.7
Albumin (g/dL)	3.7	3.2-5.2
Creatine kinase (units/L)	58	20-180
High-sensitivity troponin (ng/L)	12	0-14
pH	7.02	7.35-7.45
Anion gap	28	0-15
Laboratory testing of urine		
Urine color	Light-yellow	Yellow
Urine specific gravity	1.015	1.002-1.030
Urine protein	Trace	Negative
Urine glucose (mg/dL)	>1000	Negative
Urine ketones (mg/dL)	>100	Negative

Arterial blood gas analysis subsequently showed a pH of 7.023 (normal 7.35 to 7.45), a PaCO_2 _of 150 mmHg (normal 32.0-42.0 mmHg), a PaO_2 _of 69.5 mmHg (normal 75.0 to 100.0 mmHg), an HCO_3 _of 6.8 mmol/L (normal 20.0 to 24.0 mmol/L), and a base excess of -25.3 mmol/L (normal -2.0 to 2.0 mmol/L).

Due to volume depletion, the patient was initially unable to provide a urine sample upon admission, which delayed the diagnosis of diabetic ketoacidosis. However, once the patient's urinalysis returned positive for urine ketones, followed by a positive qualitative plasma acetone result, the diagnosis of euglycemic diabetic ketoacidosis became apparent.

The patient was started on a titrated insulin drip with intravenous 5% dextrose in 0.45% normal saline supplemented with bicarbonate. The patient was monitored with serial metabolic panels, arterial blood gas checks, and hourly glucose monitoring. On hospital day one, the patient’s acid-base status began to improve, although the anion gap did not immediately return to normal. By hospital day 2, the patient was managed in the ICU with serial labs, titrated insulin administration, vigilant electrolyte replacement, and judicious volume management until the resolution of diabetic ketoacidosis. The patient was stepped down to the medical floor on hospital day 3. The patient continued to recover uneventfully and was discharged on hospital day eight to a nursing facility. Dapagliflozin was discontinued prior to discharge.

## Discussion

Evaluation of euglycemic diabetic ketoacidosis involves a workup similar to that of classic hyperglycemic diabetic ketoacidosis, which may present a diagnostic challenge. Diagnostic criteria for classic diabetic ketoacidosis include anion gap greater than 16 mEq/L, serum glucose level greater than 250 mg/dL, blood pH less than 7.3, serum bicarbonate level less than 18 mEq/L, and an elevated serum ketone level [5-6]. Therefore, it is necessary to assess glucose, ketone production, anion gap, osmolality, serum bicarbonate, electrolytes, renal function, and pH in the acute setting [5-6]. Initial laboratory evaluation entails a comprehensive metabolic panel, serum and urine ketones, urinalysis, and arterial or venous blood gas (in patients without respiratory pathology) [5-6]. Patients may present with deranged potassium levels and should be assessed with an electrocardiogram (ECG) in addition to checking magnesium levels [5-6]. Hemoglobin A1c should be measured to assess the patient’s glycemic control [5-6]. Notably, the presence of urine ketones on dipstick analysis is not indicative of β-hydroxybutyrate, the primary ketone in diabetic ketoacidosis [5-6]. According to one study, serum β-hydroxybutyrate had a sensitivity of 98% and a specificity of 79% [5-6]. Additional workups to rule out infectious etiology may include complete blood count, blood and urine cultures, lactic acid, and chest radiography. Amylase, lipase, and transaminase levels may be obtained if co-occurring pancreatitis or non-alcoholic steatohepatitis is suspected [5-6].

Treatment of euglycemic diabetic ketoacidosis in the acute phase, according to the standard diabetic ketoacidosis protocol, is effective. As with classic diabetic ketoacidosis, the patient's volume status should be assessed, and intravenous fluids should be started at a rate of 15-20 cc/kg/hr; 0.9% saline should be used for patients with low corrected sodium, while 0.45% saline is used if corrected sodium is normal or high. At blood glucose levels below 200 mg/dL, as seen in euglycemic diabetic ketoacidosis, dextrose 5% should be added. An insulin bolus may be given at a dose of 0.1 U/kg, with continuous infusion at 0.1 U/kg/hr. Potassium should be added to fluids at a rate of 10-15 mEq/hr if the serum potassium level is between 5.2 and 3.3 mEq/L. Serum insulin should be withheld if serum potassium is less than 3.3 mEq/L and replaced at a rate of 20-30 mEq/hr. If the pH is less than 6.9, intravenous fluids are supplemented with bicarbonate. Blood gases, electrolytes, bicarbonate, anion gap, and renal function should be measured with serial labs every two to four hours. Once blood glucose returns to normal levels, serum pH rises above 7.3, and bicarbonate rises above 18 mEq/L, diabetic ketoacidosis is considered resolved [5-6].

Case reports of diabetic ketoacidosis with normal blood glucose levels have been mentioned in publications as early as 1973 [1]. Although the patients all had diabetes and were successfully treated via standard diabetic ketoacidosis protocols, the etiology of these early cases differed significantly from SGLT2 inhibitor-induced euglycemic diabetic ketoacidosis [1]. Overlapping metabolic derangements, such as alcoholic or starvation ketoacidosis co-occurring with diabetic ketoacidosis, may create the appearance of euglycemic diabetic ketoacidosis in lab studies [4,7]. Differentiating between types of ketoacidosis using ketoacid ratios and extensive laboratory workup may not be practical in the emergency or critical care setting, especially if the patient is improving with diabetic ketoacidosis treatment. According to the literature, many cases defined as euglycemic diabetic ketoacidosis, which are not attributed to SGLT2 inhibitors, may actually describe diabetic ketoacidosis with overlapping metabolic derangements [4].

SGLT2 inhibitors introduce a unique consideration: the possibility of diabetic ketoacidosis with blood glucose in the normal postprandial range [2-4,8-9]. In euglycemic diabetic ketoacidosis, SGLT2 inhibitors lower blood glucose levels by inhibiting renal glucose reabsorption. In reduced carbohydrate or caloric intake scenarios, glucagon levels rise due to inhibition of glucose transport into alpha cells via the sodium-glucose transport protein [2]. The mutual suppression of insulin occurs due to glucagon and decreased blood glucose concentration, leading to increased lipolysis and the production of ketone bodies [2-4]. Meanwhile, rising gluconeogenesis maintains blood glucose levels above the starvation range, and subsequent recovery of insulin sensitivity may further limit hyperglycemia [2-3]. Thus, while classic diabetic ketoacidosis arises from insulin deficiency, euglycemic diabetic ketoacidosis suppresses insulin activity. Coupled with the renal excretion of glucose by SGLT2 inhibitors, normal blood glucose is maintained in patients with euglycemic diabetic ketoacidosis.

Notably, a 2015 paper analyzed data from the Food and Drug Administration's Adverse Event Reporting System (FAERS) and revealed a seven-fold risk increase for diabetic ketoacidosis in type 2 diabetic patients taking SGLT2 inhibitors [10-11].

## Conclusions

The increasing use of SGLT2 inhibitors necessitates a vigilant approach to the diabetic patient with ketoacidosis. To ensure timely and effective management, healthcare providers must be aware of the potential for euglycemic diabetic ketoacidosis in patients on SGLT2 inhibitors who present with metabolic acidosis and ketonemia. This case is one of many where a euglycemic patient on an SGLT2 inhibitor presents with signs, symptoms, and laboratory results consistent with diabetic ketoacidosis. The inability to rely on hyperglycemia as a diagnostic indicator in these patients highlights the need for a cautious and thorough approach to the acidotic patient on SGLT2 inhibitors.

## References

[1] Munro JF, Campbell IW, McCuish AC, Duncan LJ (1973). Euglycaemic diabetic ketoacidosis. Br Med J.

[2] Bonner C, Kerr-Conte J, Gmyr V (2015). Inhibition of the glucose transporter SGLT2 with dapagliflozin in pancreatic alpha cells triggers glucagon secretion. Nat Med.

[3] Rosenstock J, Ferrannini E (2015). Euglycemic diabetic ketoacidosis: a predictable, detectable, and preventable safety concern with SGLT2 inhibitors. Diabetes Care.

[4] Peters AL, Buschur EO, Buse JB, Cohan P, Diner JC, Hirsch IB (2015). Euglycemic diabetic ketoacidosis: a potential complication of treatment with sodium-glucose cotransporter 2 inhibition. Diabetes Care.

[5] Arora S, Henderson SO, Long T, Menchine M (2011). Diagnostic accuracy of point-of-care testing for diabetic ketoacidosis at emergency-department triage: β-hydroxybutyrate versus the urine dipstick. Diabetes Care.

[6] Westerberg DP (2013). Diabetic ketoacidosis: evaluation and treatment. Am Fam Physician.

[7] Burgess SC (2015). Regulation of glucose metabolism in liver. International Textbook of Diabetes Mellitus, Fourth Edition.

[8] Rawla P, Vellipuram AR, Bandaru SS, Pradeep Raj J (2017). Euglycemic diabetic ketoacidosis: a diagnostic and therapeutic dilemma. Endocrinol Diabetes Metab Case Rep.

[9] Nasa P, Chaudhary S, Shrivastava PK, Singh A (2021). Euglycemic diabetic ketoacidosis: a missed diagnosis. World J Diabetes.

[10] Hsia DS, Grove O, Cefalu WT (2017). An update on sodium-glucose co-transporter-2 inhibitors for the treatment of diabetes mellitus. Curr Opin Endocrinol Diabetes Obes.

[11] Blau JE, Tella SH, Taylor SI, Rother KI (2017). Ketoacidosis associated with SGLT2 inhibitor treatment: analysis of FAERS data. Diabetes Metab Res Rev.

